# Evaluation of antigen specific recognition and cell mediated cytotoxicity by a modified lysispot assay in a rat colon carcinoma model

**DOI:** 10.1186/1756-9966-31-9

**Published:** 2012-02-01

**Authors:** Valentina Bordignon, Paola Cordiali-Fei, Monica Rinaldi, Emanuela Signori, Andrea Cottarelli, Manuela Zonfrillo, Fabrizio Ensoli, Guido Rasi, Maria Pia Fuggetta

**Affiliations:** 1 Laboratory of Clinical Pathology and Microbiology, San Gallicano Dermatologic Institute, Via Elio Chianesi, 53, 00144 Rome, Italy; 2 Institute of Translational Pharmacology (IFT), National Research Council (CNR), Via Fosso del Cavaliere 100, 00133 Rome, Italy; 3 GLURES, Academic SPINOFF Ca Foscari University of Venice, Venice, Italy

**Keywords:** LysiSpot, ELISpot, Tumor antigens, CTLs, BDIX rats, Colon cancer

## Abstract

**Background:**

Antigen-specific CD8+ cytotoxic T lymphocytes represent potent effector cells of the adaptive immune response against viruses as well as tumours. Therefore assays capable at exploring the generation and function of cytotoxic T lymphocytes represent an important objective for both clinical and experimental settings.

**Methods:**

Here we show a simple and reproducible assay for the evaluation of antigen-specific CD8+ cytotoxic T lymphocytes based on a LysiSpot technique for the simultaneous determination of antigen-specific IFN-γ production and assessment of tumor cytolysis. The assay was developed within an experimental model of colorectal carcinoma, induced by the colorectal tumor cell line DHD-K12 that induces tumors in BDIX rats and, in turn, elicits a tumor- specific immune response.

**Results:**

Using DHD-K12 cells transfected to express *Escherichia coli *β-galactosidase as target cells, and by the fine setting of spot colours detection, we have developed an in vitro assay that allows the recognition of cytotoxic T lymphocytes induced in BDIX rats as well as the assessment of anti-tumour cytotoxicity. The method highlighted that in the present experimental model the tumour antigen-specific immune response was bound to killing target cells in the proportion of 55%, while 45% of activated cells were not cytotoxic but released IFN-γ. Moreover in this model by an ELISPOT assay we demonstrated the specific recognition of a nonapeptide epitope called CSH-275 constitutionally express in DHD-K12 cells.

**Conclusions:**

The assay proved to be highly sensitive and specific, detecting even low frequencies of cytotoxic/activated cells and providing the evaluation of cytokine-expressing T cells as well as the extent of cytotoxicity against the target cells as independent functions. This assay may represent an important tool to be adopted in experimental settings including the development of vaccines or immune therapeutic strategies

## Background

A major effort in the tumour immunology research area is directed to the identification of tumor antigens for the development of specific anti-tumour immune therapies. Several putative anti-cancer vaccines have been studied in animal models through immunization with intact tumour cells, cancer-related peptides, Ag-loaded dendritic cells (DCs), different viral delivery systems as well as vaccines combined with adoptive T-cell therapy [1-3]. The enhanced anti-cancer activity, elicited by these different approaches of immunization, is mediated either by the generation of specific CD8^+ ^T cells or by an enhancement of their functional activity [4]. A number of clinical trials have indicated that anti-tumor vaccination and active immunotherapy with tumor-specific peptide vaccines represent a promising therapeutic tool against cancer. Ideally, an effective vaccine should induce specific cytolytic immune cells against molecular targets expressed only on tumor cells. On this basis, a correct and accurate detection and quantification of antigen-specific CTLs represent an essential requirement for monitoring vaccine efficacy and may provide a critical biomarker for vaccine assessment in preclinical and clinical studies on both vaccine and drug development.

While the antigen-specific T cells recognition occurs at very low frequencies in the blood, it requires the assays extremely sensitive as flow cytometry technique [5], tetramer/pentamer binding techniques [6], CD107 mobilization assay [7] or Fluorospot assays for cytokine secretion [8].

The ELISpot assay, which can detect antigen-activated T cells frequencies as low as 1/1,000,000, offers a reliable evaluation of the frequencies of these cells among peripheral blood mononuclear cells (PBMC) [9]. In fact, ELISpot assay for IFN-γ and granzyme B [10], have gained increasing popularity to measure CTL activity and are routinely used. Nevertheless, antigen-activated T cells may not always secrete the all set of their potential cytokine production [11] and conversely, cytotoxicity does not always correlate with IFN-γ secretion in bulk PBMC populations [12-14]. For this reason, few years ago has been proposed a LysiSpot assay, which is capable to detect cytotoxic T cells, and to provide an evaluation of the target cell lysis by measuring the release of a foreign marker protein [15]. In the original paper, the target tumour cells were transduced by an herpes simplex virus (HSV) amplicon vector to express *Escherichia coli *β-galactosidase (β-gal) as the marker protein.

In this study we used an experimental model of a colorectal carcinoma induced by the tumour cell line DHD-K12 in syngeneic immunocompetent BDIX rats [16]. This model, closely mimics the characteristics of human cancer (colorectal carcinoma) counterpart, being very useful to assess specific tumour immunotherapy strategies. In fact, DHD-K12 cells constitutionally express a nonapeptide epitope called CSH-275. The CSH-275 is present in tissue specimens from colorectal neoplasia but not in the normal mucosa of BDIX rats. The inoculation of CSH-275 peptide in tumour-harbouring rats induces a significant increase in CTLs activity against autologous DHD-K12 cells [17]. In addition, this nonapeptide is a major epitope identified on the Tumour Liberated Proteins (TLP) isolated from human colorectal cancer as well as in human lung and breast tumours [16-20].

Therefore, in this experimental model we adopted a modified version of the LysiSpot assay, based on a non viral transfection method to obtain ß-gal-expressing tumor target cells, combined with an IFN-γ ELISpot in a dual-colour testing, aiming at developing a method to analyze tumour specific immune responses.

Moreover in this paper we confirm that the nonapeptide epitope CSH-275 is a good marker for colorectal cancer since ex vivo lymphocytes from BDIX rats, primed with DHD-K12 are able to recognize this specific antigen.

## Methods

### Rats and tumor cells

Inbred male BDIX rats (Charles River, Calco, Italy), 8 weeks old (average weigh 220-250 g), were held for 7 days, housed in a pathogen-free animal facility and kept in accordance with European Community guidelines.

The DHD-K12 cell line (kindly obtained from Dr. F. Martin, Dijon, France), originally established from a 1,2-dimethylhydrazine-induced colon adenocarcinoma in syngeneic BDIX rats, was cultured as monolayers in DMEM supplemented with 10% heat-inactivated FCS, 2 mM L-glutamine, 100 U/ml penicillin and 100 μg/ml streptomycin at 37°C in a humidified atmosphere of 5% CO_2_. All media and supplements were obtained from Hyclone (Logan, UT). DHD-K12 cells were split 1 day before tumor challenge, detached with Cell Dissociation Solution (Sigma, St. Louis, MO), washed and diluted to the appropriate concentration in sterile PBS solution.

Following a 1-week acclimatation period and after rat anesthetization by inhalation ofO2 and 1-bromo-2-chloro-1,1,1-trifluoroethane (Sigma, St Louis, MO, USA) at 4% concentration through a vaporizer, tumours DHD-K12 cells (2 × 10^6 ^in 0.2 ml/animal) were injected s.c. in the shaved cervical region of BDIX rats.

Tumor growth (data not shown) was evaluated as previously described [16].

### Rat peripheral blood mononuclear cells

PBMC were obtained by cardiac puncture from 5 intact healthy rats, or from 5 tumor challenged rats after 30 days from DHD-K12 injection. PBMC were recovered by centrifugation through a Ficoll-Hypaque gradient (Lympholyte-H sterile solution Cederlane, Ontario, Canada), frozen in freezer medium (90% heat inactivated FBS, Euroclone, and 10% DMSO, Sigma) and kept in liquid nitrogen until employed as effector cells in the in vitro assays.

### Transfection of target cells

DHD-K12 cells employed as target cells for CTL detection were transfected by the pCMV-LacZ (kindly provided by M. Scarpa, University of Padova, Italy), containing the CMV immediate-early promoter/enhancer and the nuclear targeted β-galactosidase coding region. The pCMV-LacZ was obtained by using a commercial kit (Qiagen™ Endofree Megaprep, Qiagen S.p.A., Italy) and following the manufacturer's supplied protocol. The identity was confirmed by agarose gel electrophoresis of both uncut and restriction digested plasmid. Contamination with RNA was not observed and the majority of the plasmid was present as covalently closed circles. A lipofectamine transfection standard protocol was performed in accordance with the manufacturer's instructions (Invitrogen s.r.l, Milano, Italy) with some modifications. Briefly, 2 × 10^6 ^cells were plated in 60 mm plates in the presence of 5 ml of DMEM medium (Euroclone, Pero, Milan, Italy) with 10% FCS (Euroclone); after 24 h, the cells reached 90% confluency. Lipofectamine 2000 (25 μl) was then mixed with 10 μg of the plasmid pCMV-LacZ in 0.5 ml of DMEM and the mixture was allowed to stand at room temperature for 20 min. The transfection complex (0.5 ml) Lipofectamine 2000-DNA was added to the plate containing the cells in a volume of 5 ml of culture medium.

**Figure 1:**
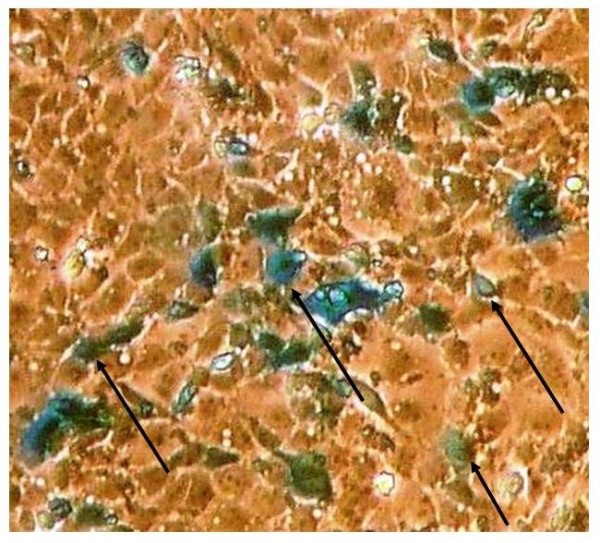
**DHD-K12 cells expressing β-gal**. DHD-K12 cells were transiently transfected with a plasmid vector expressing LacZ gene. Twenty-four hours after transfection, cells were checked for expression of β-gal through the development of blue colour. Cells expressing β-gal (mark with an arrow-head) ranged between 50% and 60% without significant cell death. The images (20x) was captured using Spot RT software version 3.0 (Diagnostic Instruments, inc) using a conventional inverted microscope.

Twenty-four hours after transfection the cells were stained using the β-Gal Staining kit (Invitrogen) to control the expression of the LacZ gene product. After removing growth medium and extensive washing with PBS, cells were fixed by 20 min of incubation with PBS containing2% formaldehyde, washed in PBS and then incubated for 6 h at 37°C with X-gal staining solution (1 mM X-gal, 5 mM potassium ferrocyanide, 2 mM MgCl_2 _in PBS). Afterwards, cells were checked under a conventional inverted fluorescence microscope to count the blue-stained, β-gal expressing cells (Figure 1). At least 200 cells were counted in four different fields. Samples with frequencies of β-gal expressing cells between 60% and 70% were used as target cells for CTL detection. No background staining was observed in DHD-K12 cells transfected with Lipofectamine 2000 without DNA (negative control).

### ELISpot assay for the analysis of IFN-γ producing cells

The enumeration of individual cells producing IFN-γ, was performed by a commercially available immunospot assay kit (PVDF Rat IFN-γ ELISpot Kit, Euroclone, Pero, MI, Italy) following the manufacturer's instructions with some modifications. Briefly, polyvinylidene fluoride microtiter plates (MAIP S45 10, Millipore Sunnyvale, CA, USA) were coated overnight at 4°C with capture MoAb anti-IFN-γ, dissolved in sterile PBS, 100 μl/well. Ab-coated plates were then washed and incubated 2 h at room temperature with complete medium (RPMI 1640, 10% FBS, 1% Penicillin-Sptreptomycin-L-Glutamine; GIBCO-BRL, UK) to prevent non-specific protein binding.

Cryopreserved PBMC from control or tumour harbouring rats were thawed and cultured in triplicate wells (2 × 10^5^/well) with different concentrations (10-4-2-1 μg/ml) of CSH-275 peptide (gently provided by Cell Essentials, Boston, MA) in a humidified atmosphere with 5% CO_2 _at 37°C. Control wells containing PBMC with medium alone or with PHA (10 μg/ml, Sigma, Saint Louis, MO, USA) were also tested.

After 20 h of incubation, cells were lysed with ice-cold distilled water and removed by rinsing (four times) with PBS/0.05% Tween^® ^20 (Sigma, St Louis, MO, USA). After 90 min incubation with abiotynilated anti-IFN-γ detection MoAb, diluted in PBS with 1% bovine serum albumin (BSA, fraction V, Sigma, St Louis, MO, USA), Streptavidin alkaline phosphatase conjugate (diluted in sterile PBS with 1% BSA) was added to the wells for 45 min at 37°C in the dark. The plates were then washed and refilled with a ready-to-use BCIP/NBT solution. Blue spots were let to develop for up to 30 min at r.t. in the dark. Plates were then washed with distilled water to stop the reaction and allowed to dry overnight. Spots were counted by an Automated ImmunoSpot Image Analyzer Software (AELVIS Tecnologies, TEMA-Ricerca, Italy). The stimulation index (S.I.) was expressed by the ratio between the number of spots per 2 × 10^5 ^PBMC plated with antigen and those detected in control wells [21].

### The cytotoxic assay

The specific cytotoxic activity of PBMC from DHD-K12-inoculated rats (Immune, I) or control (Non Immune NI) was tested by using a Promega CytoTox 96 kit (Promega, Corporation Madison, WI). This colorimetric assay quantitatively measures the release of lactate dehydrogenase (LDH), a stable cytosolic enzyme. Briefly, target cells were incubated in 96-well round bottom plates with effector cells in 10:1, 5:1, 2,5:1 and 1,25:1 effector/target cell ratios for 4 h at 37°C. All samples were run in quadruplicate. Spontaneous release of effector or target cells was controlled by separate incubation of the respective population. At the end of incubation, the cells were lysed and centrifuged. 100 μl aliquot of each well was transferred into another 96 well plate and 100 μl of freshly "LDH substrate solution" was added to each well. The plates were incubated, light-protected, at room temperature for additional 10 min, and the reaction was stopped by the addition of acetic acid 1 M. The resulting light absorbance was measured in a microplate reader (Multiskan EX Labsystem) at 490 nm. The percentage of cytotoxic activity was calculated according to the following formula:

$$\text{cytotoxicity}\;=\frac{\text{Eexp}-\text{Esp}-\text{Tsp}\%}{\text{Ttot}-\text{Tsp}}\times 100$$

where Eexp is the experimental LDH release of co-cultured effector and target cells, Esp and Tsp express the spontaneous released LDH of the effector and target cell alone, respectively, and Ttot is the maximum LDH amount of target cells.

### The LysiSpot assay

The LysiSpot assay was set by a procedure similar to that of the ELISpot assay, with some modifications. In brief, polyvinylidene fluoride microtiter plates (MAIP S45 10, Millipore Sunnyvale, CA, USA) were coated with capture MoAb against β-gal (from mouse fractionated ascites fluid, clone G4644 Sigma, Saint Louis, Missouri, USA) diluted at 12 μg/ml in PBS with 1% BSA. DHD-K12 target cells were plated 5 h after transfection at 1-4 × 10^4^/well with effector cells (PBMC at 2 × 10^5^/well) in complete RPMI medium and cultured for 16 h at 37°C in a 5% CO_2_. Biotinylated anti-β-gal detection MoAb (clone GAL 13 Sigma) diluted at 2 ug/ml in PBS with 1% BSA was added in a volume of 100 μl/well. After 90 min, avidin-horseradish peroxidase was added to the plates and incubated for 1 h incubation at r.t. (Pierce Biotechnology, Rockford, IL, USA). Plates were then washed and incubated with AEC-chromogen solution (BD Biosciences, Belgium) until red spots were clearly visible.

### Dual-colour LysiSpot assay

Plates were coated with a mixture of capture MoAbs against β-gal and IFN-γ. Effector and target cells were prepared as in the LysiSpot assay (see above). After 16 h of incubation, Biotinylated anti-IFN-γ detection MoAb was added to the plates, followed by streptavidin-alkaline phosphatase conjugate. After washing, a 30 min, incubation with an unrelated biotinylated MoAb (we used MoAb anti-IL-4 diluted in RPMI) was performed to block any free streptavidin binding sites. Afterwards, the biotinylated β-gal detection MoAb was added to the plates, followed by avidin-horseradish peroxidase conjugate. The plates were then incubated sequentially with the peroxidase substrate AEC and the phosphatase substrate BCIP/NBT to develop respectively red and blue spots.

The dual-colour settings programme (AELVIS Technologies, Software-version 4.2 Reader, TEMA-Ricerca, Italy) allowed to count the spots separately for three different colours. After setting up the limits the spots were sorted into three groups: pure red (β-gal) or blue spots (IFN-γ) and violet spots (concomitant IFN-γ and ß-gal release). Wells with DHD-K12 target cells or PBMC cultured alone were considered as controls and the corresponding spots were subtracted from the number of spots obtained in the co-cultures.

### Statistical analysis

The results were analyzed by non parametric Mann Whitney *t *test, using GraphPad Prism version 5.00 for Windows (GraphPad Software, San Diego California USA, http://www.graphpad.com).

## Results

### Target cells

Transfected tumour cells DHD-K12 showing β-gal expression ranged between 50% and 60% in different experiments (Figure 1). No background staining was observed in cells transfected with Lipofectamine 2000 without DNA, performed as negative control (not shown).

### IFN-γ release

**Figure 2:**
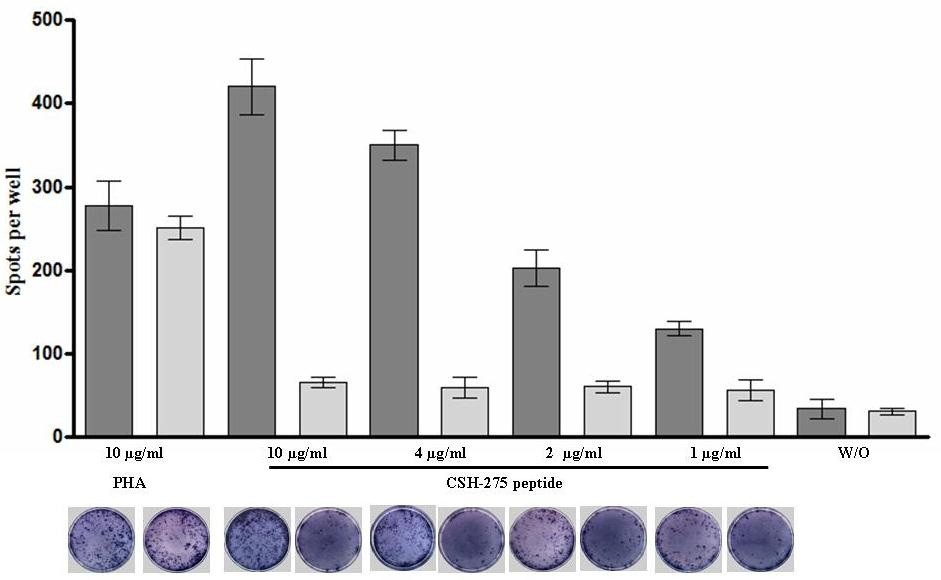
**IFN-γ release**. IFN-γ-ELISpot results from three different experiments, expressed as number of spots per well (mean ± SD), showed the immune-response of DHD-K12-inoculated rats (dark grey) against CSH-275 peptide. No effect was produced on PBMC from control rats (light grey). Increasing concentration of peptide yielded an increasing numbers of IFN-γ producing PBMC. Under each histogram there is the corresponding image illustrative of blue spots. As negative contros we showed the non stimulated PBMC (W/O).

The specific T-cell recognition of the CSH-275 peptide antigen was evaluated in vitro through the analysis of the IFN-γ release. The stimulation of PBMC from DHD-K12-inoculated rats, using different concentration of CSH-275 peptide, induced the production of IFN-γ in a dose-dependent manner. The response induced by concentrations of 4-10 μg/ml of the peptide antigen was even higher than that induced by the mitogen. PBMC from control rat did not respond to the CSH-275 peptide, while they had an IFN-γ response to mitogen similar to that observed in DHD-K12-inoculated rats. These findings confirmed that DHD-K12-inoculated rats develop a specific immune response against the CSH-275 peptide expressed on DHD-K12 cells [16], and that such response is measurable in vitro by the ELISpot assay for IFN-γ. In Figure 2 are reported the mean stimulation indexes obtained in three different experiments.

### Cytotoxic activity

**Figure 3:**
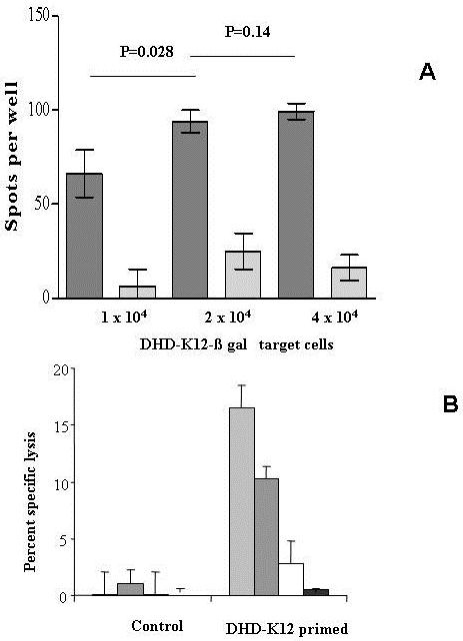
**Panel **A **- LysiSpot assay**. LysiSpot assay results, expressed as net number of spots per well (spots from wells containing only target cells were subtracted), from four different experiments (mean ± SD). Increasing numbers of target cells were plated in short term cultures with effector cells (2 × 10^5^/well PBMC). Spots were the imprint of β-gal, released by the transfected DHD-K12 target cells after lysis. Cytotoxic activity of PBMC from DHD-K12-inoculated rats or control rats are represented by dark and light grey respectively. Panel **B **- LDH-Cytotoxicity assay. Cytotoxic activity expressed as percent of specific lysis (mean ± SD) of DHD-K12 target cells from PBMC of intact (control) or DHD-K12-inoculated rats (Primed) evaluated by Promega CytoTox 96 kit. Concentration ratio of effector and target cells was 10:1 (light grey), 5:1 (dark grey), 2.5:1 (white), 1.25:1 (black) and corresponding respectively to 2 × 10^4^,1 × 10^4^, 5 × 10^3^, 2.5 × 10^3 ^of DHD-K12 target cells.

DHD-K12-inoculated rats developed aspecific cytolytic T cell response towards tumor cells. In Figure 3A are depicted the histograms representing the number of spots corresponding to the release of β-gal from lysed target cells. In these experimental settings, 2 × 10^5^/well PBMC were plated in the presence of different number of DHD-K12 β-gal transfected target cells. Only PBMC from DHD-K12 inoculated rats developed a cytotoxic activity against the tumour, while control rats were unable to recognize and kill target cells. The number of induced spots was dose-dependent and increased in the presence of higher number of target cells up to 2 × 10^4^. Peak concentration corresponded to 2 × 10^4 ^target cells. Higher concentrations did not lead to a significant increase in spots (*P *= 0.14).

To further demonstrate the in vitro specific cytotoxicity of PBMC from intact or DHD-K12-inoculated rats against DHD-K12 cell line we utilized a colorimetric assay (CytoTox 96 kit Promega) that quantitatively measures the release of lactate dehydrogenase (LDH) from killed tumor cells. In Figure 3B the results, expressed as percent of specific lysis confirm, at comparable effector: target ratio used in Lysispot, the specific cytotoxic activity against DHD-K12 tumor cell line.

### Cytotoxicity and IFN-γ secretion evaluated by the dual-colour LysiSpot assay

The dual-colour assay allowed to determine both the induction of cytotoxic effects in association with the production of IFN-γ in response to the specific recognition of the tumor cells.

**Figure 4:**
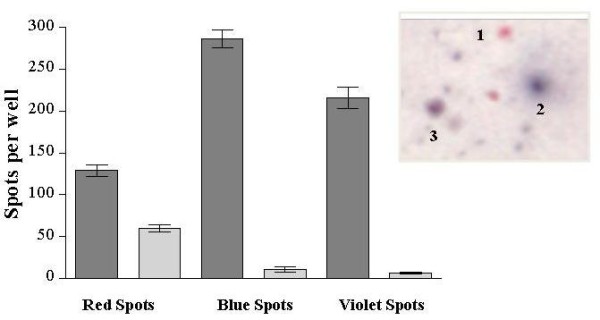
**Dual-colour LysiSpot assay**. Dual-colour LysiSpot assay results from three different experiments (mean ± SD), expressed as net number of spots per well (spots from control wells containing only target cells or PBMC cultured alone were subtracted from the spots counted in the cocultures. DHD-K12 transfected cells (2 × 10^4^/well) were cocultured with 2 × 10^5^/well PBMC. The panel shows the image of the different spots left on the wells at the end of assay: 1) pure red spots indicate cell lysis by IFN-γ non-producing cells; 2) pure blue spots indicate IFN-γ secreting cells; 3) violet spots indicate cell lysis by IFN-γ producing cells. Dark and light grey bars represent number of spots from DHD-K12-inoculated rats or from control rats respectively.

DHD-K12 β-gal transfected cells (2 × 10^4^) were cultured with 2 × 10^5 ^PBMC from control or tumor harbouring rats. Trough the combined analysis of the spots of different colours, a differential counts of the number of lysed cells (pure red spots), the number of PBMC secreting IFN-γ (pure blue spots) and the number of cells that simultaneously secreted IFN-γ and lysed the targets (violet spots combining both colours) was allowed. The histograms depicted in Figure 4, represent the results of three different experiments and show that IFN-γ secretion and cytotoxicity are distinct CTLs functions that can be independently regulated. Therefore, in our experimental conditions, 55% of the overall immune activated cells developed a full lytic activity and a large portion of these cells (65%) also released IFN-γ. The remaining 45% produced IFN-γ but were not cytotoxic.

## Discussion

The development of sensitive assays to assess specific T cell responses against cancer represents a key tool for both experimental and clinical immunology as well as in the pre-clinical and clinical settings [9,22,23].

In recent years, the increase in the understanding the biology of tumor cells and the identification of tumor antigens capable to elicit potent and effective T cell immune responses, opened an avenue of possibilities for the design of specific vaccination strategies based on the use of peptide antigens [24,25]. Is therefore of utmost relevance the development of assays that can provide qualitative and quantitative measurement of the anti-tumour immune responses.

Several techniques for immune monitoring of specific T-cell responses are now available including assays which provide information about the specific T cell recognition of cancer antigens, irrespective of their functional activity, such as those based on the use of tetramers [26], assays aimed at detecting T-cell precursors by amplifying cells that proliferate in response to the antigenic stimulation [27], as well as assays that measure the secretion of a particular cytokine [28] All these test do not provide information about the anti-tumour lytic activity of the immune cells [9,28]. On the other hand, the assessment of cytotoxicity, is generally measured on the basis of the Chromium or Europium release assay, Such cytotoxicity assays measure the percentage of targets lysed by a bulk population of effectors, but they do not provide any information about the frequency of cyotoxic T cells.

The biologic relevance of these methods is therefore limited to the specific information about cytokine secretion, extent of cell-mediated cytotoxicity and/or proliferation in response to tumour antigens. Nevertheless, antigen-activated T cells do not necessarily secrete the same set of cytokines, neither cytotoxicity always correlates with cytokine secretion in a bulk T cell population [12,14,29].

It is well recognised that activated CD8+ T cells mediate their functions by secretion of different cytokines, including IFN-γ, that initiate a "lytic program" ending with a direct perforin-mediated transfer of lytic enzymes (granzyme) capable of inducing apoptosis in target cells [10,30-32].

As previously demonstrated in vitro by Snyder JE et al. [15] the cytotoxic activity and IFN-γ production by CTLs are independent functions which may follow different regulatory pathways. In fact, not all CD8+ T cells function as "killer" cells. Indeed, during the acute phase of a CD8+ T-cell response, IFN-γ production, cytotoxicity, and proliferation appeared as independently regulated in cancer and infections [15,33,34].

The simultaneous determination of the different functions exerted by T cells can offer a valuable tool for ex vivo analysis of the immune response against cancer as well as infections, but also in assessing autoimmune diseases as well as to identify correlates of immune protection exploitable for therapeutic strategies based on vaccine development.

The assay we developed is based on a dual-colour LysiSpot method aimed at measuring the extent of the recognition of tumour cells by CTLs, as elicited in a rat model harbouring a colorectal tumour induced by the DHD-K12 cell line. In this assay the simultaneous determination of the different functions exerted by T cells can offer a valuable tool for ex vivo analysis of the immune response against cancer as well as furnish a base to evaluate the number and function of lytic effector cell.

DHD-K12 cells naturally express a tumour-associated antigen that induces specific cytotoxic responses in immune competent syngeneic animals [16,17]. The synthetic nonapeptide antigen, CSH-275, was previously used in a vaccination protocol and gave proof of the induction of an antitumour activity as elicited by the vaccination [17]. By the ELISPOT assay illustrated in Figure 1 we have further demonstrated the specific recognition of this nonapeptide, epitope constitutionally express in DHD-K12 cells

In the present study, the DHD-K12 cell line was transiently transfected, using a pCMV-LacZ vector containing the nuclear-targeted β-gal coding region. This method permits to easily "mark" [35] the tumour cell line. We chose to use the plasmid DNA- Lipofectamine complex to introduce a gene expressing a marker protein because this methodology with non-viral vectors, either plasmids or siRNAs, efficiently transfects human colon cancer cells [36-39] as well primary neurons. In the latter, optimized protocols gives transfection efficiencies of 20-30%, a great improvement compared with less than 3% previously reported [40].

Non-viral vectors have been receiving increasing attention, since they are safer and cheaper, and can be produced easily in large quantities. A recent study comparatively examined a panel of non-viral gene transfer systems in several cells of different origins, including human colorectal carcinoma, and in human primary cells [41]. In this work, the authors evaluated the requirements for successful transfection and the potential for optimization of transfection efficiency. Their results indicate that this high efficiency methodology can be sufficiently optimized to offer a feasible approach for gene delivery into a wide range of cells, including human tumor cells [41].

Using a commercially available IFN-γ ELISpot assay, we confirmed an antigen-specific, dose-dependent, IFN-γ release by PBMC isolated from rats when primed with DHD-K12 cells.

The dual-colour assay was developed by combining an IFN-γ ELISpot assay, a LysiSpot assay, and β-gal transfection of the target cells. This assay allowed us to detect simultaneously the lysis of tumour target cells and the identification of CTLs producing IFN-γ. The use of a dual-colour software programme, allowed to count separately the spots of three different colours, thus overcoming the reported difficulty in discerning the difference in the colours of the spots previously described The LysiSpot was performed with a number of target cells high enough to virtually allow all CTLs present in the culture to find the target, however respecting the limit of an acceptable background level of positive spots. The assessment of effector/target cells ratio was determined in preliminary experiments (data not shown) to ensure that all the key parameters to assess T cell cytotoxicity were optimized.

The method highlighted that in the present experimental model the tumour antigen-specific immune response was bound to killing target cells in the proportion of 55%, while 45% of activated cells were not cytotoxic but released IFN-γ. Those cells could represent an incomplete stage of differentiation toward fully developed effector cells [42].

DHD-K12 cells naturally express a tumour-associated antigen that induces specific cytotoxic responses in immune competent syngeneic animals [16,17]. The synthetic nonapeptide antigen, CSH-275, was previously used in a vaccination protocol and gave proof of the induction of an antitumour activity as elicited by the vaccination [17]. These data demonstrate that CSH-275 is full recognized by ex vivo lymphocytes from DHD-K12 primed rats and since CSH-275 is a major epitope identified on the TLP (Tumour Liberated Proteins) isolated from human lung, colon and breast cancer [18-20] it is evident the importance of this antigen as a potential target for new diagnostic and/or therapeutic approaches to human cancer.

## Conclusions

In this study we show a reproducible and easy technique capable of measuring even low frequencies of antigen-specific cytolytic cells against tumour, and provided further evidence of the multiple aspects of the different regulatory pathways governing the induction of cytolytic mechanisms.

The proposed lysispot assay, and this rat colon carcinoma model, could be used to evaluate the specific cell mediated immunity and or cytochine production in preclinical study, pharmacological treatment and development of immune intervention.

## Competing interests

There are no competing interests (political, personal, religious, ideological, academic, intellectual, commercial or any other) to declare in relation to this manuscript by all authors.

## Authors' contributions

VB, AC, PCF carried out the immunoassays and participated in the design of the study and performed the statistical analysis. MR and ES carried out the transfection protocol. MZ supplied the cells from the animal model. VB, GR PCF FE helped to draft the manuscript. MPF conceived of the study, and participated in its design and coordination and helped to draft the manuscript. All authors read and approved the final manuscript.
